# Sensitive, Highly Multiplexed Sequencing of Microhaplotypes From the *Plasmodium falciparum* Heterozygome

**DOI:** 10.1093/infdis/jiaa527

**Published:** 2020-08-25

**Authors:** Sofonias K Tessema, Nicholas J Hathaway, Noam B Teyssier, Maxwell Murphy, Anna Chen, Ozkan Aydemir, Elias M Duarte, Wilson Simone, James Colborn, Francisco Saute, Emily Crawford, Pedro Aide, Jeffrey A Bailey, Bryan Greenhouse

**Affiliations:** 1 EPPIcenter Research Program, Division of HIV, Infectious Diseases, and Global Medicine, Department of Medicine, University of California, San Francisco, California, USA; 2 Department of Medicine, University of Massachusetts Medical School, Worcester, Massachusetts, USA; 3 Department of Pathology and Laboratory Medicine, Brown University, Providence, Rhode Island, USA; 4 Centro de Investigação em Saúde de Manhiça, Manhiça, Mozambique; 5 Clinton Health Access Initiative, Maputo, Mozambique; 6 Chan Zuckerberg Biohub, San Francisco, California, USA

**Keywords:** malaria, *Plasmodium falciparum*, molecular epidemiology, microhaplotype, multiplex PCR, targeted amplicon sequencing, whole genome sequencing, complexity of infection

## Abstract

**Background:**

Targeted next-generation sequencing offers the potential for consistent, deep coverage of information-rich genomic regions to characterize polyclonal *Plasmodium falciparum* infections. However, methods to identify and sequence these genomic regions are currently limited.

**Methods:**

A bioinformatic pipeline and multiplex methods were developed to identify and simultaneously sequence 100 targets and applied to dried blood spot (DBS) controls and field isolates from Mozambique. For comparison, whole-genome sequencing data were generated for the same controls.

**Results:**

Using publicly available genomes, 4465 high-diversity genomic regions suited for targeted sequencing were identified, representing the *P. falciparum* heterozygome. For this study, 93 microhaplotypes with high diversity (median expected heterozygosity = 0.7) were selected along with 7 drug resistance loci. The sequencing method achieved very high coverage (median 99%), specificity (99.8%), and sensitivity (90% for haplotypes with 5% within sample frequency in dried blood spots with 100 parasites/µL). In silico analyses revealed that microhaplotypes provided much higher resolution to discriminate related from unrelated polyclonal infections than biallelic single-nucleotide polymorphism barcodes.

**Conclusions:**

The bioinformatic and laboratory methods outlined here provide a flexible tool for efficient, low-cost, high-throughput interrogation of the *P. falciparum* genome, and can be tailored to simultaneously address multiple questions of interest in various epidemiological settings.

## Teaser

A novel bioinformatics pipeline was created to select and characterize the most diverse and tractable short-range sequences (microhaplotypes) in the *Plasmodium falciparum* genome, followed by a robust polymerase chain reaction–based multiplexing method to simultaneously amplify and sequence these microhaplotypes.

Malaria genomics has been applied to generate actionable data to inform control and elimination efforts, for example, tracking the spread of drug resistance and evaluating the response to vaccine candidates [1–3]. To realize the full potential of genomics for understanding malaria transmission, an ideal genotyping method would seek to maximize discrimination between infections, including polyclonal, low-density infections often encountered in endemic settings. Traditional genotyping methods such as typing of length polymorphisms [4], microsatellites [5, 6] and single-nucleotide polymorphisms (SNPs) [7–9] have been extensively used to characterize malaria transmission. However, technical and biological constraints limit the scalability and discriminatory resolution of these methods, particularly when infections are polyclonal [10, 11]. Typing of microsatellites and length polymorphisms suffer from difficulties in standardizing laboratory protocols, allele calling and reporting, and detection of minority clones [12]. To address challenges in throughput and standardization, several SNP barcoding approaches were developed to evaluate parasite diversity and population structure and to estimate transmission dynamics [7–9, 11, 13]. However, SNP-based methods have limited discriminatory power to compare polyclonal infections, which represent the majority of the parasite population in many places in sub-Saharan Africa, including areas of low transmission [14, 15].

Recent advances in next-generation sequencing (NGS) allow targeted deep sequencing of short, highly variable regions with numerous alleles (microhaplotypes) [16, 17], predominantly composed of 3 or more SNPs, allowing for detailed characterization of the ensemble of parasites in an infection. Most applications of these methods to date have targeted one or a few genomic loci to provide information on drug resistance, composition of infections, or selection [18–25]. Extending these methods to numerous genetically diverse loci offers the potential for high-resolution comparisons of infections at a population level [17]. To this end, there have recently been efforts to multiplex large numbers of loci that reflect overall patterns of *P. falciparum* diversity [26, 27].

With thousands of whole-genome sequencing (WGS) data available [28, 29], it is now possible to establish an optimal set of multiallelic targets to interrogate the *P. falciparum* genome using NGS. However, bioinformatics pipelines to identify informative targets are currently lacking. Furthermore, finding sensitive and high-throughput laboratory methods for targeted sequencing of these markers in a cost-effective manner remains a major challenge. In this study, we describe the initial evaluation of a bioinformatic pipeline to identify high-value *P. falciparum* microhaplotypes and a robust polymerase chain reaction (PCR)–based laboratory method that allows sequencing of hundreds of these microhaplotypes in a single reaction. This study evaluates the performance of the laboratory method and the information content of a selection of approximately 100 microhaplotypes across a range of in silico analyses, mixture controls, and field samples.

## METHODS

### Microhaplotype Selection Pipeline

Tandem repeats longer than 50 bp within the *P. falciparum* 3D7 reference genome were determined using tandem-repeat-finder [30]. Windows of 200 bp every 100 bp were created between tandem repeats. Per-base coverage and the fraction of proper pairs (ie, the proportion of pairs with both mates mapped with normal insert sizes in the proper orientation) were calculated from the raw Illumina data of 13 reference genomes [31]. Windows were kept if they were within 1 standard deviation from the average base-pair coverage of the genome and if the proper-pair fraction was >0.85 in 11 of the 13 genomes. Windows with dinucleotide repeats or homopolymers longer than 10 bp were excluded. The 3D7 sequences from these windows were then blasted against the 13 genomes with at least 75% identity and 95% coverage using LASTZ [32]. Windows were kept if they mapped once to all 13 genomes, had no length variation >3 bases, average GC content >15%, and sequences in at least 3 of the 13 genomes were unique. Local haplotype assembly was run on these final windows in WGS from 4054 field samples and 33 laboratory isolates (Supplementary Table 1).

### Mock and Field Dried Blood Spot Samples

Mock dried blood spot (DBS) samples were prepared as previously described [33]. In brief, synchronized *P. falciparum* parasites were mixed at different proportions with uninfected human whole blood to obtain a range of parasite densities (10, 100, 1000, and 10 000 parasites/μL of blood) (Supplementary Figure 1). Field DBS samples were collected from southern Mozambique from febrile malaria cases. This study was approved by the institutional review boards at the University of California, San Francisco and the Manhiça Health Research Centre, Mozambique. DBS samples were stored at –20°C until processing. DNA was extracted from a single 6-mm hole-punch using a modified Tween–Chelex protocol as previously described [33].

### Multiplexed Amplicon Sequencing

Primers were designed for 100 selected genomic regions of the microhaplotypes and drug resistance markers using the CleanPlex algorithm (Paragon Genomics). Amplification of the 200-plex oligo pool was performed with some modifications of the CleanPlex protocol (Paragon Genomics) (Supplementary Methods). Samples were bead purified, quantified, and pooled. The final library was bead purified, assessed for quality, and sequenced with 150 bp paired-end clusters on a NextSeq instrument (Illumina). The targeted amplicon data were analyzed using SeekDeep (version 2.6.6) [34] (Supplementary Methods).

### Selective Whole-Genome Amplification and WGS

Selective whole-genome amplification and WGS were performed following a previously optimized protocol [33]. After demultiplexing, reads were aligned to the *P. falciparum* 3D7 reference genome (version 3) with BWA-MEM software [35]. Variants were called using the Genome Analysis Toolkit Best Practices [36].

### Simulations to Evaluate Genetic Relatedness

Population-level allele frequencies were calculated for 91 microhaplotypes and variant loci across Pf3k samples from Ghana, which had the largest number of publicly available WGS. Simulated genomes were created with a single multinomial draw given allele frequencies across all loci. Relatedness between genomes was simulated parameterizing a Bernoulli draw by the expected relatedness over the number of loci, masking the selected loci, and making both genome pairs equivalent at each masked locus. Relatedness was calculated by converting each locus to a boolean vector, concatenating all loci in a sample to a single vector, then calculating Jaccard distance between sample pairs.

## RESULTS

### Heterozygome of *P. falciparum*: Identification of High-Diversity Genomic Regions

**Figure 1.**
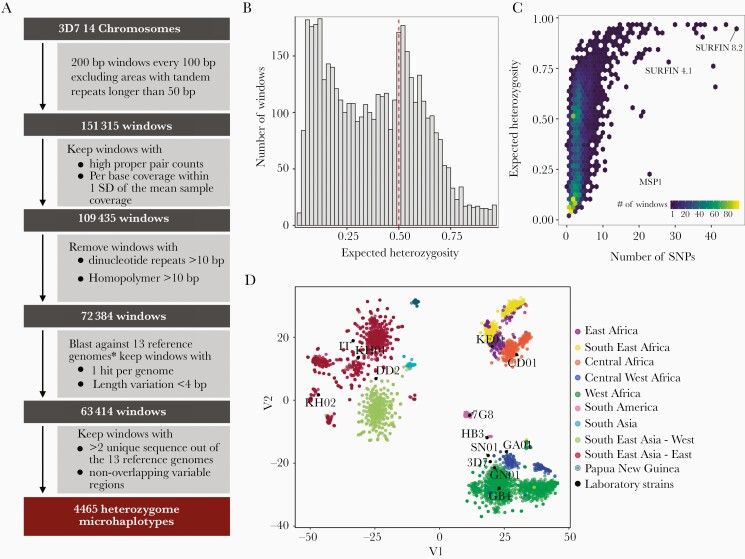
Characterization of the heterozygome of *Plasmodium falciparum* in global parasite populations. *A*, Bioinformatics workflow for the identification of the heterozygome. *Thirteen high-quality assembled and annotated genomes of *P. falciparum* isolates [31]. The variable region covered was determined as the first position to the last position within the window with a single-nucleotide polymorphism (SNP) or indel ≥0.5%. The window with the highest number of samples was kept when variable regions overlapped. *B*, Distribution of expected heterozygosity of microhaplotypes (n = 4465) in global parasite populations (n = 4054 isolates), showing high genetic diversity in a substantial number of microhaplotypes. *C*, Distribution of the number of SNPs per microhaplotype, showing the relationship between the number of SNPs and expected heterozygosity. *D*, Population structure of the global *P. falciparum* parasite population inferred from the microhaplotypes visualized using tSNE as implemented in the Rtsne package [42] with 25 000 iterations, a perplexity parameter of 100, and a trade-off θ of 0.5.

A custom pipeline was developed to perform a genome-wide scan of *P. falciparum* to identify short genomic regions that exhibit high population-level genetic diversity, here termed “heterozygome” windows (Figure 1A). We created windows of 200 bp in between long tandem repeats and blasted them against 13 high-quality assembled genomes of *P. falciparum* isolates [31]. Windows suitable for downstream PCR/sequencing (ie, no homopolymers/dinucleotide repeats longer than 10 bp, or length variation >3 bp) were kept if they uniquely mapped to all 13 genomes with ≥70% identity. Out of these 63 414 windows, 4465 windows with ≥3/13 unique haplotypes were retained as the heterozygome. Population genetic measures were calculated for the heterozygome by running local haplotype reconstruction for each window on 4054 field samples with publicly available WGS [28, 37–41] (Supplementary Table 1).

A third of the microhaplotypes in the heterozygome had high expected heterozygosity (H_E_ > 0.5 [35%]) in the global parasite population, and 356 had H_E_ > 0.7 (Figure 1B). Heterozygosity was correlated with the number of unique haplotypes in the 13 reference genomes used (Spearman ρ = 0.5; *P* < .001, Supplementary Figure 7), confirming the utility of this initial filtering approach. Geographically, the highest diversity was observed in West and Central West Africa (Supplementary Table 2). More than half (55%) of the microhaplotypes were composed of at least 3 SNPs (Figure 1C) and 846 microhaplotypes had at least 5 SNPs. Heterozygome microhaplotypes were able to accurately represent geographic structure of the global parasite population (Figure 1D).

### Selected Microhaplotypes for Multiplexed Sequencing

**Figure 2.**
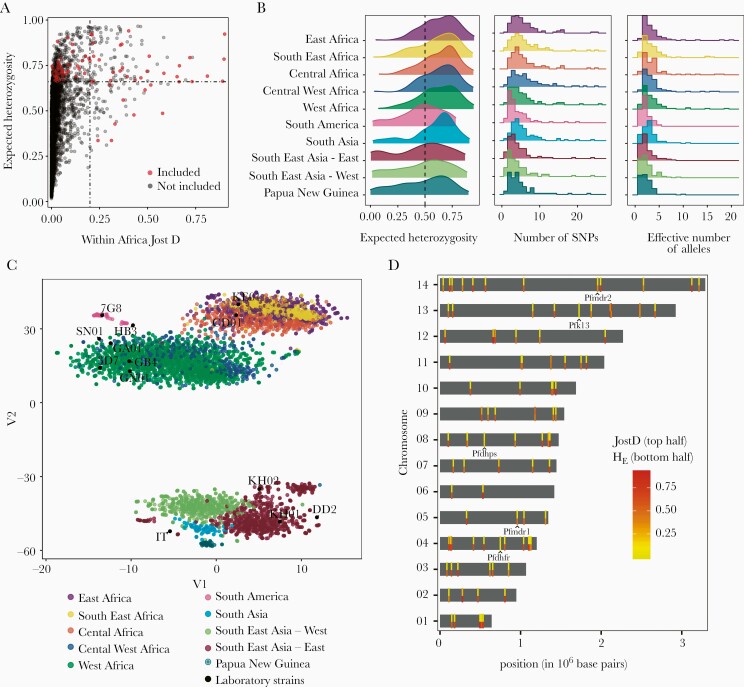
Overview of identified and selected microhaplotype loci (n = 93) and drug resistance markers (n = 7). *A*, Relationship between expected heterozygosity (H_E_) and within-Africa genetic differentiation (Jost D) for selected microhaplotypes. Microhaplotype loci were screened for identifiable primer sites before primer design. *B*, Distribution of H_E_, number of single-nucleotide polymorphism (SNPs), and the number of effective alleles of the selected microhaplotype loci in different malaria-endemic regions. *C*, Geographic clustering of global parasite populations inferred from the selected microhaplotypes visualized using tSNE as implemented in the Rtsne package [42] with 25 000 iterations, a perplexity parameter of 100, and a trade-off θ of 0.5. *D*, Chromosomal location of microhaplotypes. The mean of within-Africa genetic differentiation (top half) and mean H_E_ (bottom half) are shown by colored bars (see Supplementary Figure 6 and Supplementary Tables 2 and 3). The molecular markers of drug resistance included in this panel are also indicated.

From the 4465 microhaplotypes, the 150 most diverse (expected heterozygosity) and differentiated (Jost D ≥ 0.21) within Africa were combined with 11 molecular markers of drug resistance for primer design for 2 × 150 Illumina sequencing. From primer design, 7 markers of drug resistance had workable primers and the top 93 microhaplotypes with primers were selected for a total of 100 loci (Figure 2A). The selected microhaplotypes were genetically diverse in all malaria-endemic regions, with a median heterozygosity ranging from 0.48 in South America to 0.67 in Central Africa. On average, there were 5 (interquartile range [IQR], 3–7) SNPs and 3.4 (IQR, 2.8–4.0) effective alleles in the selected microhaplotypes (Figure 2B). A subset of the microhaplotype loci was highly differentiated between malaria-endemic regions and between countries within a region (Supplementary Figure 8), leading to strong population structure (Figure 2C). The selected microhaplotype loci were distributed throughout the 14 chromosomes of the parasite (median, 6 [range, 2–11] loci per chromosome; mean interlocus distance, 137 075 base pairs [8.06326 centimorgans]) (Figure 2D, Supplementary Table 4). Five targets (t2, t21, t64, t76, t93) had variable amplification and were excluded after additional analysis revealed primer sites had SNPs that prevented certain laboratory strains from amplifying (Supplementary Figure 5 and Supplementary Table 4).

### Multiplexed Targeted Sequencing of Microhaplotype and Drug Resistance Markers

**Figure 3.**
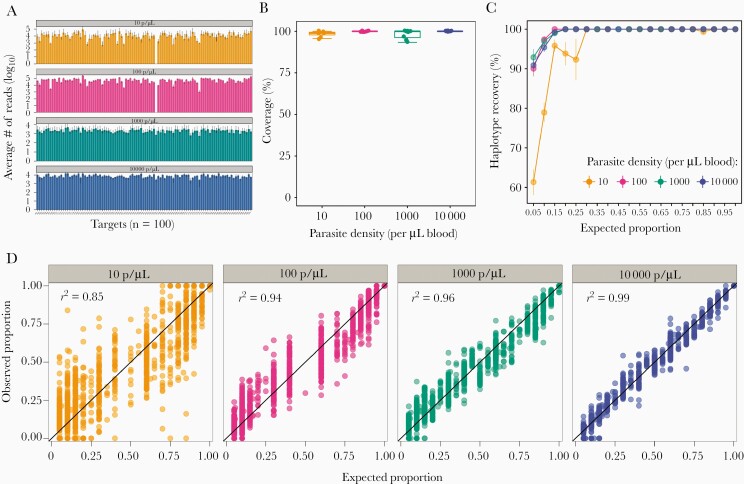
Coverage and sensitivity of multiplexed targeted sequencing of microhaplotypes (n = 93) and drug resistance markers (n = 7) on control samples. *A*, Average number of reads per target per sample. The median (bars) and interquartile range (error bars) are shown. *B*, Boxplot summarizing the coverage of microhaplotype loci and drug resistance targets by parasite density. Coverage was determined based on the number of targets with 250 or more reads. *C*, Sensitivity of the assay for the detection of haplotypes at different proportions, stratified by total sample parasite density. Microhaplotype recovery was calculated as the number of observed haplotypes matching to the expected microhaplotypes divided by the total number of expected haplotypes. *D*, Correlation of expected and observed within-sample proportions of microhaplotypes by parasite density is shown.

A 2-step multiplex PCR-based assay was optimized using CleanPlex chemistry (Paragon Genomics) for evenness of coverage and detection of minority clones. The assay was evaluated on a range of DBS controls containing known proportions of laboratory strains (Supplementary Figure 1). A high level of uniformity in coverage was achieved across different parasite densities (Figure 3A), with detection of alleles ranging from 93% to 100% in samples down to 10 parasites/µL (Figure 3B). The assay demonstrated high specificity regardless of the proportions of strains in the mixture (true positive rate, 99.8%). The sensitivity of the method was high across a wide range of parasite densities (Figure 3C). For example, at a within-sample haplotype proportion of 0.05, an average sensitivity of 61% was observed for 10 parasites/µL total parasite density and 90% for 100 parasites/µL, indicating the suitability of the method for the detection of minority clones even in low-density infections (Figure 3C). Finally, the high correlation between the observed and expected haplotype proportions indicates the potential of this method for accurate quantification of within-host proportions of strains in mixed infections (Figure 3D).

### Targeted Sequencing Provides Substantially Better Detection of Microhaplotypes Than WGS

Compared with targeted sequencing, WGS provides broader evaluation of the genome but at the expense of sequence depth in specific regions of interest. To compare the sensitivity of targeted vs WGS for detection of microhaplotype loci, WGS data were generated from the same DBS mixture controls, using selective whole-genome amplification, at high depth and coverage (Supplementary Figure 2). From these WGS data, reads spanning the entire variable regions of microhaplotypes were extracted to obtain unambiguous sequences. The extracted data were analyzed to evaluate coverage, detection of minority clones, and quantification of strains. In contrast to results from targeted sequencing, microhaplotypes extracted from WGS data had poor coverage and sensitivity for the detection of minority alleles, even at the highest parasite densities evaluated (Supplementary Figure 3).

### Microhaplotypes Provide Accurate Estimation of Complexity of Infection and Genetic Relatedness

**Figure 4.**
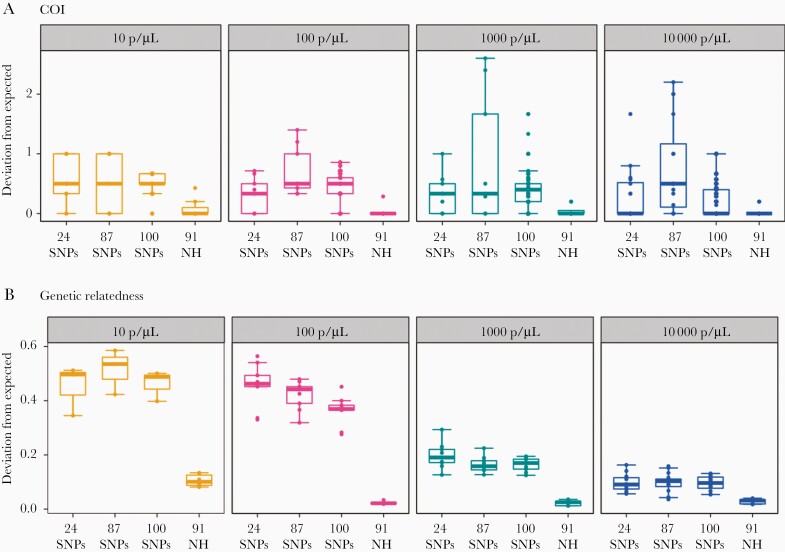
Comparisons of marker sets for complexity of infection (COI) and pairwise genetic relatedness. *A*, The deviation of observed COI from the expected number of strains in the mixture controls. COI was determined using THE REAL McCOIL [43] for single-nucleotide polymorphisms (SNPs) and the number of unique haplotypes per sample for microhaplotypes; the majority of incorrect calls for all methods were under predicted COIs. *B*, The deviation of observed genetic relatedness from that expected. The observed pairwise genetic relatedness was determined using Jaccard distance and is compared with the expected genetic relatedness between the control samples. Deviation was calculated as mean absolute error. Twenty-four SNP barcodes, 87 SNPs, and 100 high-coverage and high-diversity SNPs were extracted from the whole-genome sequencing data. Ninety-one high-quality microhaplotype loci (MH) were used for these analyses.

To compare the accuracy of complexity of infection (COI) estimation and genetic relatedness between infections by microhaplotype and SNP-based approaches, we extracted molecular SNP barcodes (n = 24 [7]), SpotMalaria SNPs (n = 87 of the 101 SpotMalaria SNPs [9, 29]) and 100 high-coverage and high-diversity SNPs with >0.2 minor allele frequency from the WGS data generated on the controls. Compared to biallelic SNPs, targeted sequencing of microhaplotypes consistently achieved substantially more accurate estimation of COI and, even more dramatically, pairwise genetic relatedness at all parasite densities, indicating the high discriminatory power of microhaplotypes for the measurement of within-host diversity and genetic relatedness between infections (Figure 4). These findings are consistent with the high genetic diversity of the selected microhaplotype loci and the high sensitivity of the assay for detecting minority clones.

### Microhaplotypes Provide Higher Discriminatory Power Than Individual SNPs for Estimating Genetic Relatedness of Polyclonal Infections

**Figure 5.**
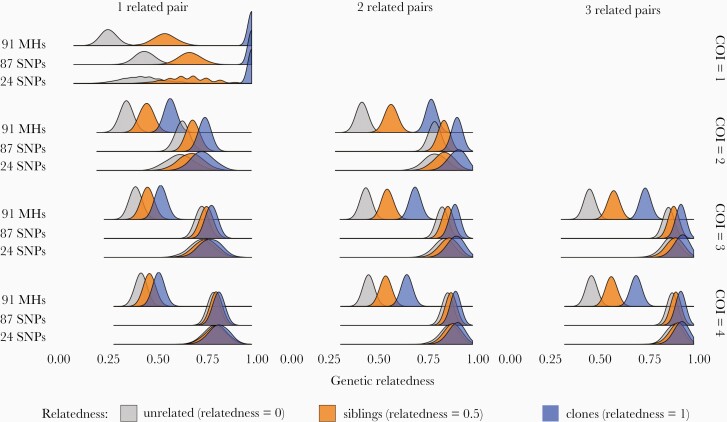
In silico comparison of genetic relatedness estimated from multiallelic and biallelic loci in polyclonal infections. Peaks represent distributions of genetic distance obtained from 2000 simulated infection pairs for each combination of complexity of infection (COI), relatedness, and set of loci. Separation between the peaks demonstrates discriminatory power among unrelated, sibling, and clones of parasites using 91 microhaplotypes (MHs), 87 SpotMalaria single-nucleotide polymorphisms (SNPs), and 24 SNP barcodes.

Polyclonal infections are common in many endemic areas, making it difficult to estimate genetic relatedness between infections. To evaluate the discriminatory power of microhaplotypes vs SNPs, a simple simulation of infection pairs with varying numbers of clones and related parasites was performed. Perfect detection of alleles was assumed, to isolate the information content of the loci from the sensitivity of the laboratory method. For monoclonal infections, all methods performed similarly and were able to easily discriminate unrelated parasites from siblings and clones (Figure 5). However, in polyclonal infections, microhaplotypes provided higher genetic resolution to discriminate related from unrelated infections across all scenarios evaluated. We note that the simulation framework and distance metric used here were intentionally straightforward in order to convey a high-level comparison of the information content of the various sets of loci, and do not represent a comprehensive, quantitative evaluation using methods directly tailored at inferring ancestry.

### Validation of Multiplexed Amplicon Sequencing in Field Samples

**Figure 6.**
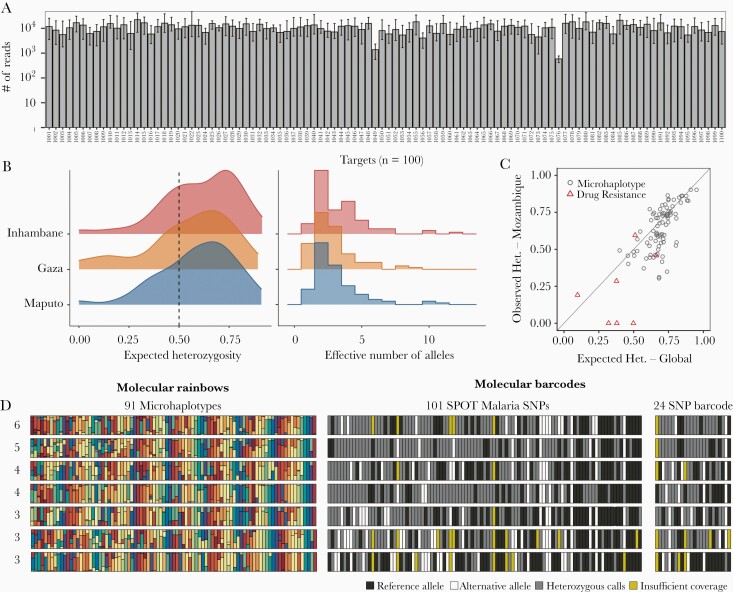
Coverage of multiplexing assay on selected microhaplotypes (n = 93) and drug resistance markers (n = 7) on field samples. *A*, Average number of reads per target per sample. The median (bars) and interquartile range (error bars) are shown for 82 field samples. *B*, Distribution of expected heterozygosity and the number of effective alleles in 3 provinces of southern Mozambique. *C*, Correlation of expected heterozygosity of microhaplotypes in the global population and the observed heterozygosity in the Mozambique samples. *D*, Visualization of microhaplotypes and single-nucleotide polymorphism barcodes in polyclonal infections. Rows are samples and columns represent loci. For the microhaplotypes, each allele is represented by a unique color at that locus and the size of the stacked bar is proportional to the within-host proportion of the allele, generating a “molecular rainbow.” Abbreviations: Het., heterozygosity; SNP, single-nucleotide polymorphism.

Eighty-two *P. falciparum* DBS samples from southern Mozambique were genotyped using the microhaplotype panel. Similar to the control samples, a high level of uniformity in the average number of reads per target (Figure 6A) and a coverage of >95% was achieved in samples with at least 10 parasites/µL (Supplementary Figure 9*A*). Analyses of replicate DBS samples extracted and processed independently showed the reproducibility of the assay for the detection and quantification of haplotypes in monoclonal and polyclonal samples (Supplementary Figure 9*B*). The majority (68%) of the infections were polyclonal with a mean COI of 2.3 (Supplementary Figure 9*C*). The selected microhaplotypes were also diverse in the 3 provinces of southern Mozambique (average heterozygosity, 0.6; average effective alleles, 2.6), and 72 of the 93 microhaplotypes had H_E_ > 0.5 in at least 1 of the 3 provinces (Figure 6B). There was strong correlation (*r*^2^ = 0.74; *P* < .001) between the heterozygosity of the microhaplotypes in the global parasite population and the observed heterozygosity in Mozambique (Figure 6C), confirming the validity of the pipeline to identify high-diversity genomic regions even in those countries that did not have publicly available WGS for the target selection. Visualization of microhaplotypes and biallelic SNPs (obtained from WGS data) on the same polyclonal samples illustrated the potential of microhaplotypes to better characterize these infections, given the large number of heterozygous SNP calls (Figure 6D). Analyses of drug resistance targets that were multiplexed in this assay showed absence of known resistance-associated K13 mutations in this population.

## DISCUSSION

In this study, a bespoke bioinformatic pipeline was developed and validated to identify thousands of globally diverse, multiallelic microhaplotypes throughout the *P. falciparum* genome—the heterozygome. Using multiplex PCR, we were able to simultaneously sequence 100 heterozygome microhaplotypes and key drug resistance targets in a single reaction with consistent, deep coverage, detecting and quantifying minority alleles in DBS samples down to 10 parasites/µL of blood and outperforming whole-genome sequences obtained from 50 times more total reads. Data from laboratory controls and in silico analyses indicate that this approach allows for better estimation of genetic diversity within and genetic relatedness between polyclonal samples when compared with panels of SNPs, making it a promising tool for studying the transmission dynamics of *P. falciparum*.

Measuring genetic relatedness between malaria infections is a fundamental step toward translating genomic data into operationally relevant information on transmission dynamics and tracking parasite flow [10, 44, 45]. The utility of genetic data in evaluating relatedness is largely driven by the diversity of the markers used, with greater diversity generally giving better resolution. When comparing individual parasites to each other, a sufficient number (eg, a few hundred) of moderately diverse SNPs can theoretically provide sufficient resolution for comparisons, because combinations of many SNPs occurring within an individual parasite may be informative [44]. In practice, the ability of SNP panels to measure meaningful differences in relatedness is limited in many settings because a large proportion of infections are polyclonal, reducing the multiplicative benefit of numerous markers since phased combinations belonging to individual parasites are not directly observed. In such settings, the incremental value of increasing the diversity of each locus beyond that obtained from biallelic SNPs becomes greater and may be necessary to obtain meaningful information. Intuitively, this is because in the absence of phasing, comparisons between polyclonal infections are often reduced to determining whether or not alleles are shared at each locus; when all possible alleles at a locus are present (2 in the case of most SNPs), there will always be alleles shared with all other infections. As a result, many studies have only compared monoclonal infections, limiting power and potentially introducing bias into analyses. Multiallelic markers such as microsatellites have been used to overcome these limitations in the past but are cumbersome; microhaplotypes accomplish a similar objective using current sequencing technology by unambiguously phasing multiple SNPs that are close enough to be sequenced in a single read [16, 17, 46]. As demonstrated here, the combinations of SNPs within properly selected microhaplotypes can provide substantial diversity, dramatically outperforming independent SNPs in the ability to estimate relatedness between polyclonal infections. Furthermore, we have shown that deep sequencing of microhaplotypes provides sensitive detection and accurate quantification of minority alleles, potentially allowing for in silico phasing of microhaplotypes in mixed infections and allowing for even higher resolution comparisons.

While we show the application of this approach using approximately 100 microhaplotypes as an example, the number and composition of markers can be tailored to specific questions of interest and target populations. For example, to understand within-host diversity and genetic relatedness on a timescale that is relevant to directing and evaluating interventions (eg, driven by recombination events occurring over months to a few years), high-diversity microhaplotypes should provide the required resolution. Based on our analysis, many of these loci are diverse across multiple geographic settings. If the goal is to perform spatial assignment of infections, microhaplotypes with high genetic differentiation between infections from relevant geographic regions should be considered. These may be more specific to particular contexts, as signals of genetic differentiation are more varied geographically and at different spatial scales. Thus, the availability of globally diverse windows across different transmission zones should allow the development of a core panel, with the potential to add, for example, a subset of highly differentiated microhaplotypes for geographic regions of interest or evenly distributed windows across the genome for Identity by decent analysis. It should be noted that many of the microhaplotypes identified herein may be under balancing or directional selection. As such, they may not be appropriate for analyses that mandate neutral markers [47]. If neutrality is preferred but not strictly required, the statistical power of having diverse markers must be balanced against this preference. Another consideration is that this study evaluated microhaplotypes that can be sequenced with 150 bp paired-end reads; improvements in long-read targeted sequencing may additionally allow the consideration of longer microhaplotypes or minihaplotypes (~10 kb) for increased genetic resolution and greater flexibility to incorporate complex and diverse regions of the genome [48].

This analysis has identified a set of generally informative markers and has begun to evaluate the utility of using microhaplotypes for high-resolution genotyping of low-density and polyclonal infections. However, recognizing the full potential of genetic data generated from this approach will require additional improvements in downstream analysis. Few analytical tools directly address the challenges of incorporating multiallelic data or evaluation of polyclonal infections; even fewer encompass both. For example, to our knowledge there are no established methods for calculating basic statistics such as population-level allele frequency or formally calculating genetic distance between polyclonal infections using multiallelic markers, forcing the use of ad hoc metrics such as those used here and in prior analyses [14]. Near-term investment in developing these tools will be necessary to take full advantage of the rich data generated from *P. falciparum* microhaplotypes in translating genomic data into meaningful epidemiologic intelligence. The development of appropriate analytical frameworks will also facilitate the rational selection of informative microhaplotype panels, for example, determining how many and which microhaplotypes provide adequate resolution to answer a set of questions in a range of epidemiologic settings. Availability of the appropriate amplicon panels and calibrated tools will bring us much closer to mapping epidemiologic and genetic data onto operationally relevant measures such as changes in transmission intensity, evaluation of the impact of interventions, accurate classification of local and imported infections, and evaluation of transmission chains [49, 50].

Incorporating genomics into malaria surveillance in endemic settings will not be trivial, but the availability of efficient and information-rich strategies for generating genomic data from routinely collected field samples, such as that evaluated here, is an important step toward this goal. The ability to simultaneously obtain deep sequence data from targets informing drug resistance and multiple aspects of transmission epidemiology should allow for streamlining and standardization of laboratory and bioinformatic pipelines, allowing these technologies to be transferred more rapidly to endemic settings. Additional components not evaluated here, such as species identification, an expanded number of candidate and validated molecular markers of drug resistance, and markers of diagnostic resistance (eg, *hrp2/3* deletions), would enable a larger set of use cases to be addressed simultaneously. With such a panel and appropriate analytical tools, an integrated approach to genomic surveillance allowing for routine generation of these valuable data in the areas where they are needed most may be closer to becoming a reality.

## Supplementary Data

Supplementary materials are available at *The Journal of Infectious Diseases* online. Consisting of data provided by the authors to benefit the reader, the posted materials are not copyedited and are the sole responsibility of the authors, so questions or comments should be addressed to the corresponding author.

jiaa527_suppl_Supplementary_Table_S1

jiaa527_suppl_Supplementary_Table_S2

jiaa527_suppl_Supplementary_Table_S3

jiaa527_suppl_Supplementary_Table_S4

jiaa527_suppl_Supplementary_Figures

jiaa527_suppl_Supplementary_Methods

## Notes

***Acknowledgments.*** The authors thank the participants in the Mozambique studies; the participants’ parents and guardians; and the field study team who performed sample and data collection. This publication uses data from the MalariaGEN *Plasmodium falciparum* Community Project (www.malariagen.net/projects/p-falciparum-community-project).

***Disclaimer.*** The funders of the study had no role in the study design, data collection, data interpretation, or writing of the manuscript. The authors had final responsibility for the decision to submit for publication.

***Financial support.*** This work was supported by the Bill & Melinda Gates Foundation (award number OPP1132226) and by the Chan Zuckerberg Biohub. MalariaGEN’s genome sequencing was performed by the Wellcome Trust Sanger Institute. The Community Projects are coordinated by the MalariaGEN Resource Centre with funding from the Wellcome Trust (grant numbers 098051 and 090770).

***Potential conflicts of interest.*** All other authors report no potential conflicts of interest.

Presented in part: 68th Annual Meeting of the American Society of Tropical Medicine and Hygiene, National Harbor, Maryland, 20–24 November 2019.
