# Supplementary material for: Sensitive, Highly Multiplexed Sequencing of Microhaplotypes From the Plasmodium falciparum Heterozygome
Source: J Infect Dis. 2020 Aug 25;225(7):1227–37. doi: 10.1093/infdis/jiaa527 (PMC8974853; doi:10.1093/infdis/jiaa527)
Supplement: jiaa527_suppl_Supplementary_Figures [file jiaa527_suppl_supplementary_figures.docx]

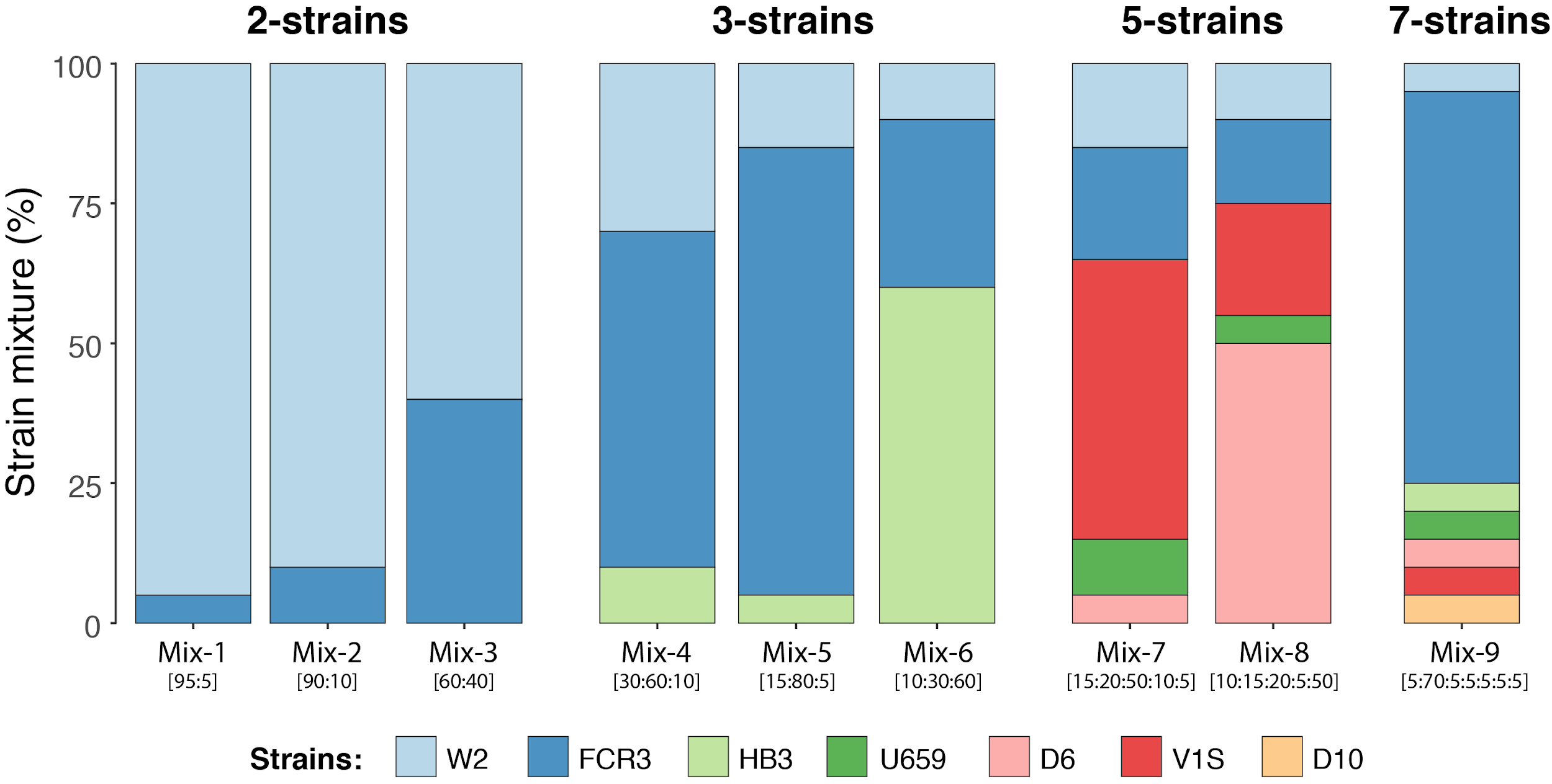


**Supplementary Figure 1. Strain composition of mock mixture controls.** Different numbers of strains were mixed at different proportions to simulate polyclonal infections.


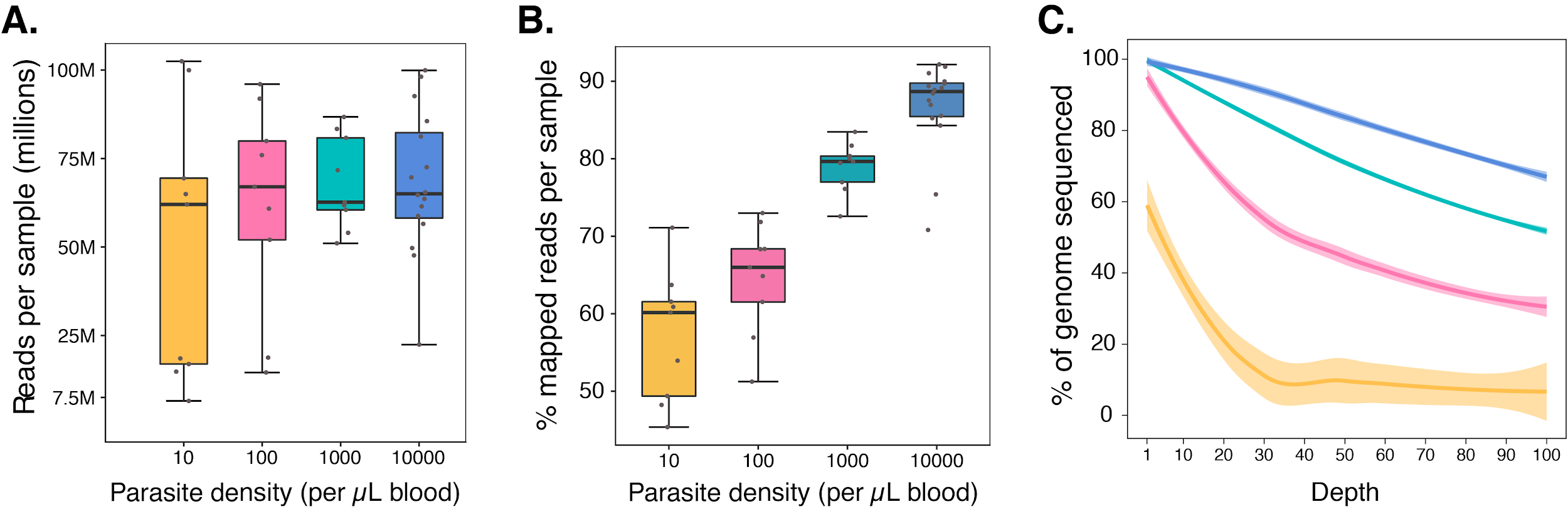


**Supplementary Figure 2. Coverage of whole genome sequencing of mixture control samples. A.** Box plot showing the total number of reads sequenced by parasite density. **B.** Boxplot showing the proportion of reads mapped to the core *P. falciparum* genome. **C.** The percentage of the core *P. falciparum* genome covered by a minimum read depth is shown; colors correspond to parasite density in panels A and B.


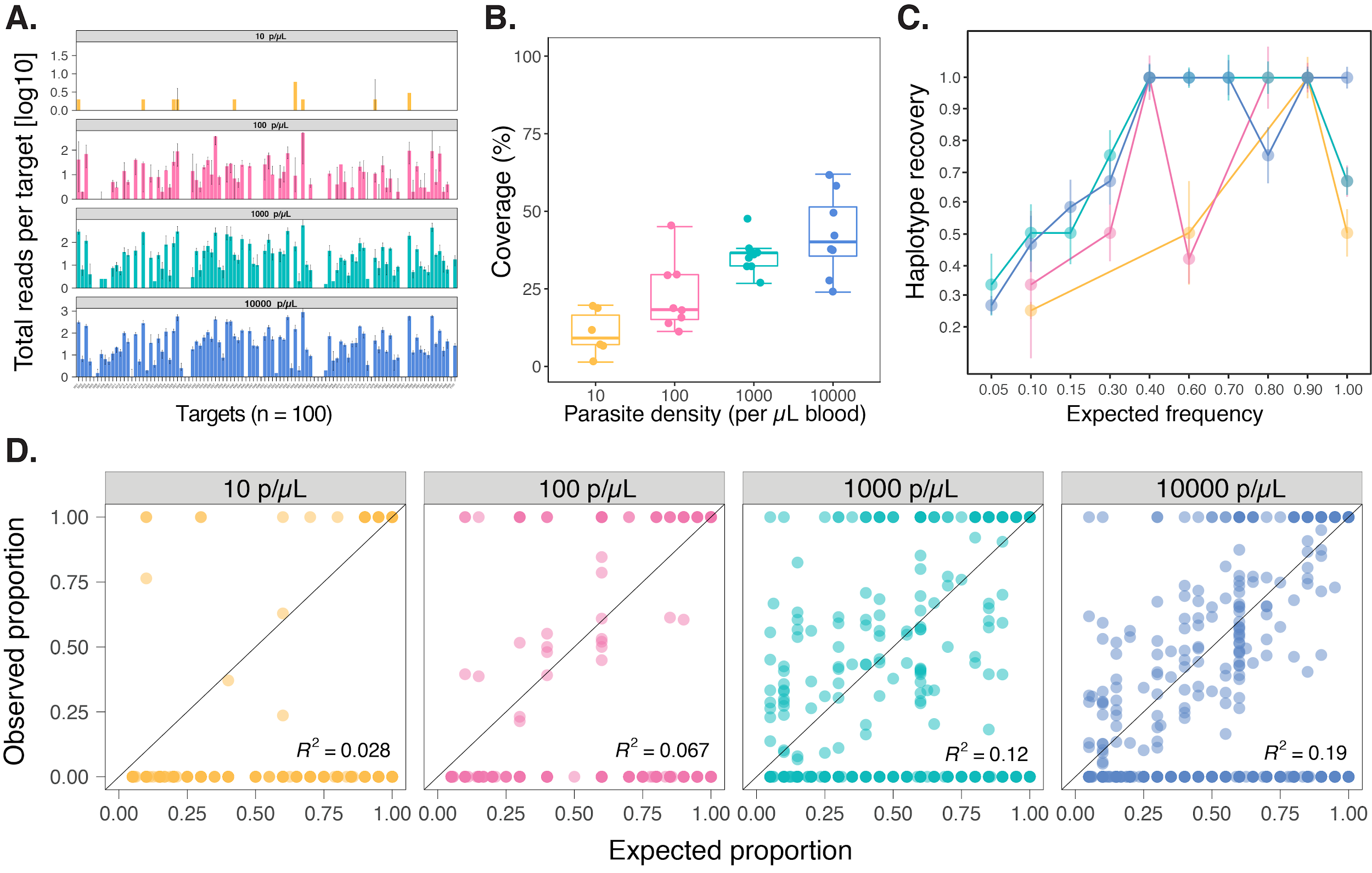
 **Supplementary figure 3. Coverage and sensitivity of microhaplotype sequences extracted from WGS of mixture control samples. A.** Average number of fully spanning reads per microhaplotype. The median (bars) and interquartile range (error bars) are shown. **B.** Boxplot summarizing the coverage of full-length microhaplotype loci extracted from WGS data is shown by parasite density. **C.** Microhaplotype recovery in WGS extracted reads shown by parasite density. **D.** Correlation of expected and observed frequencies of microhaplotypes by parasite density is shown.


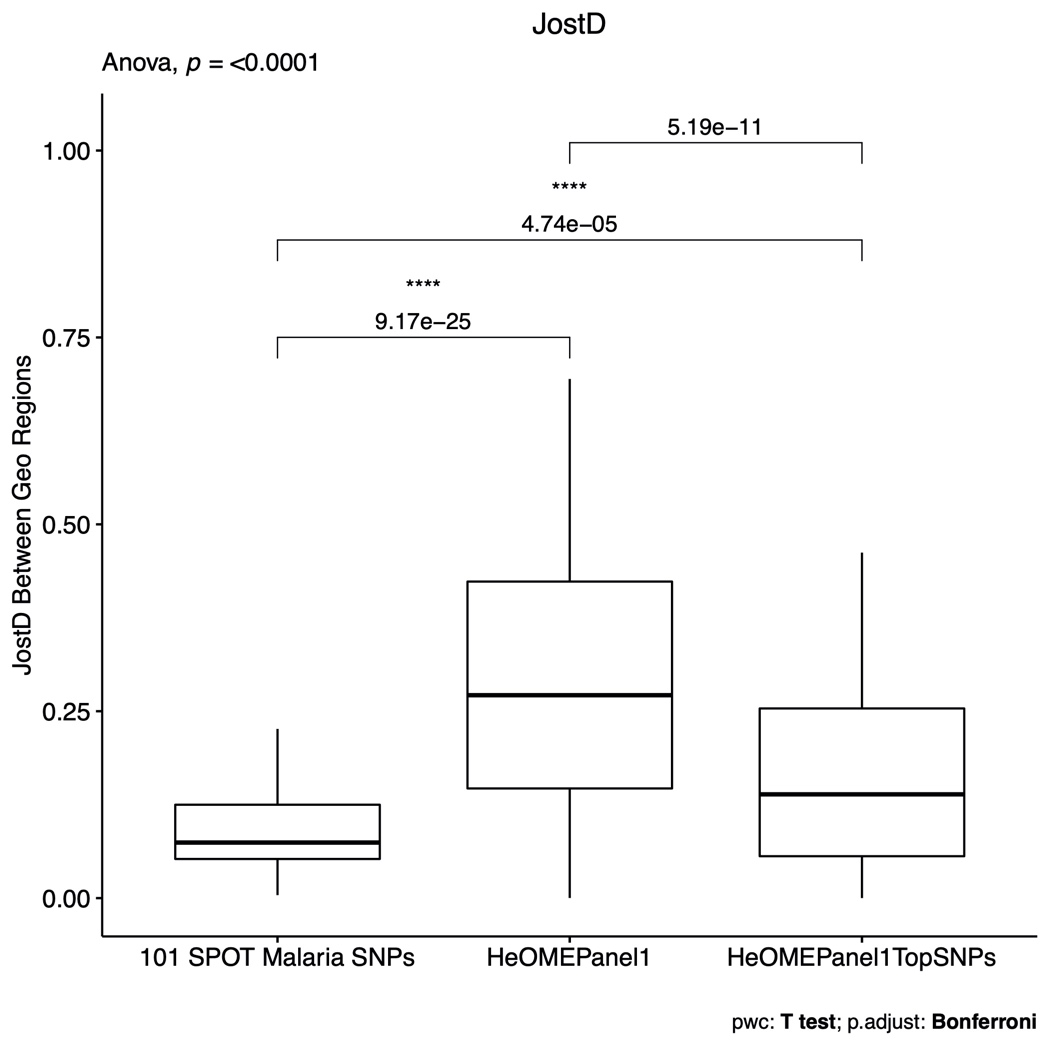


**Supplementary figure 4. Comparison Between JostD for Microhaplotype Panel vs 101 SPOT Malaria barcode.** JostD was calculated between geographic regions in **Figure 2D** using the publicly available data for the 101 SNPs SPOT Malaria barcode, the HeOME panel (93 microhaplotypes), and the most diverse SNP from each target within the panel and the average JostD was compared between the sets. The HeOME panel had a significantly higher JostD between regions than either the SNP barcode or the top SNPs from each target which shows the power of using microhaplotypes with several SNPs compared to individual SNPs and that this isn’t driven by the most diverse SNP in each target but by the combination of the SNPS within target.


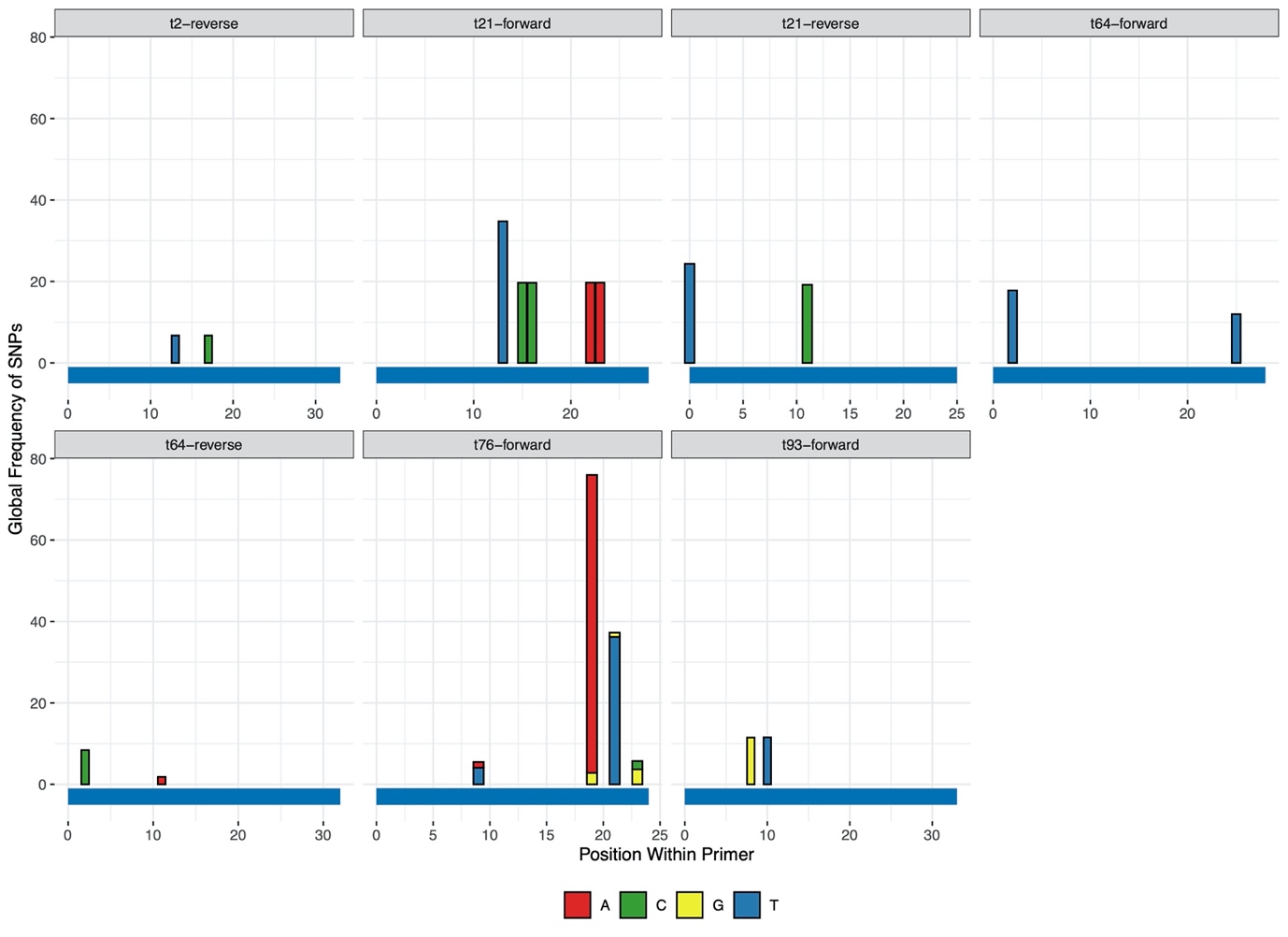


**Supplementary figure 5. SNP frequencies within target primers.**

This figure shows the global frequency of SNPs within the primers of several of the primer targets for the amplicon panel created. The original VCF used to create the primers for these targets did not have these SNPs due to various filtering parameters in the variant calling pipeline and an updated VCF will be used for future primer design. The SNPs within these primers prevented specific strains from ever amplifying within control mixtures demonstrating the importance of accurate SNP data when designing primers.


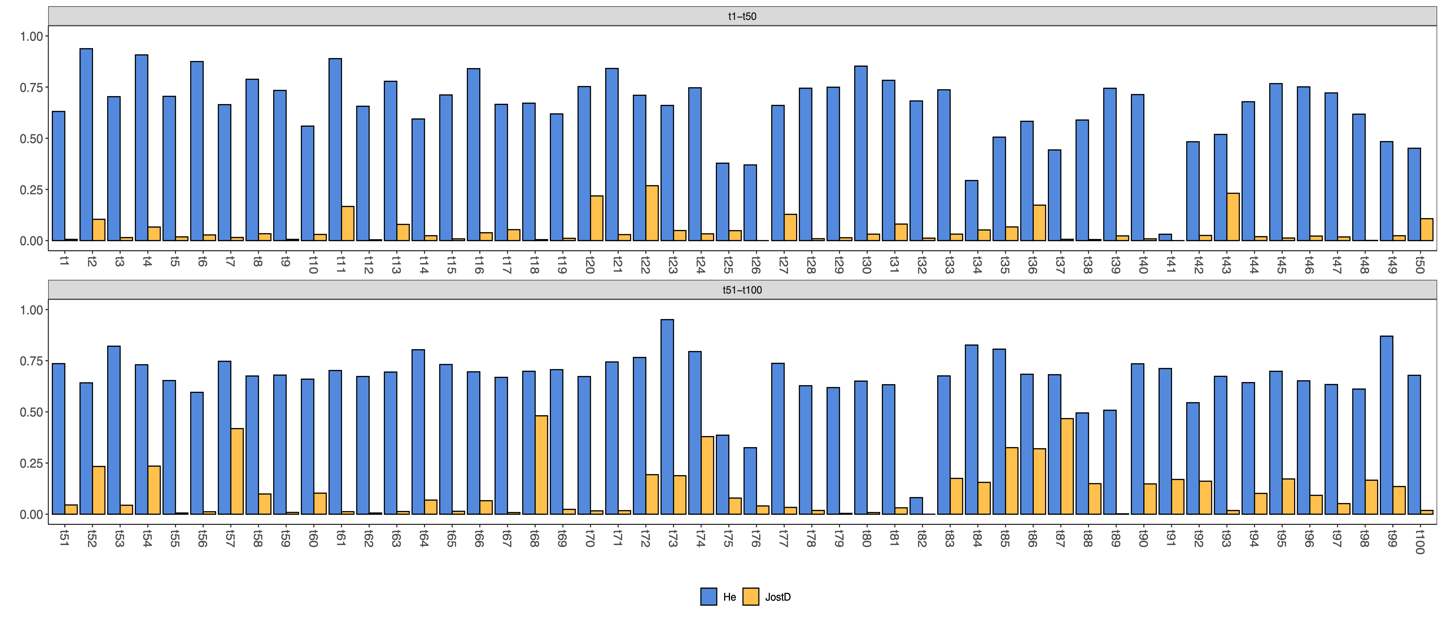


**Supplementary figure 6. Within-Africa expected heterozygosity (He) and Jost’s D for targets**

Within-Africa expected heterozygosity and Jost’s D from Figure 2E for the 100 targets.

**
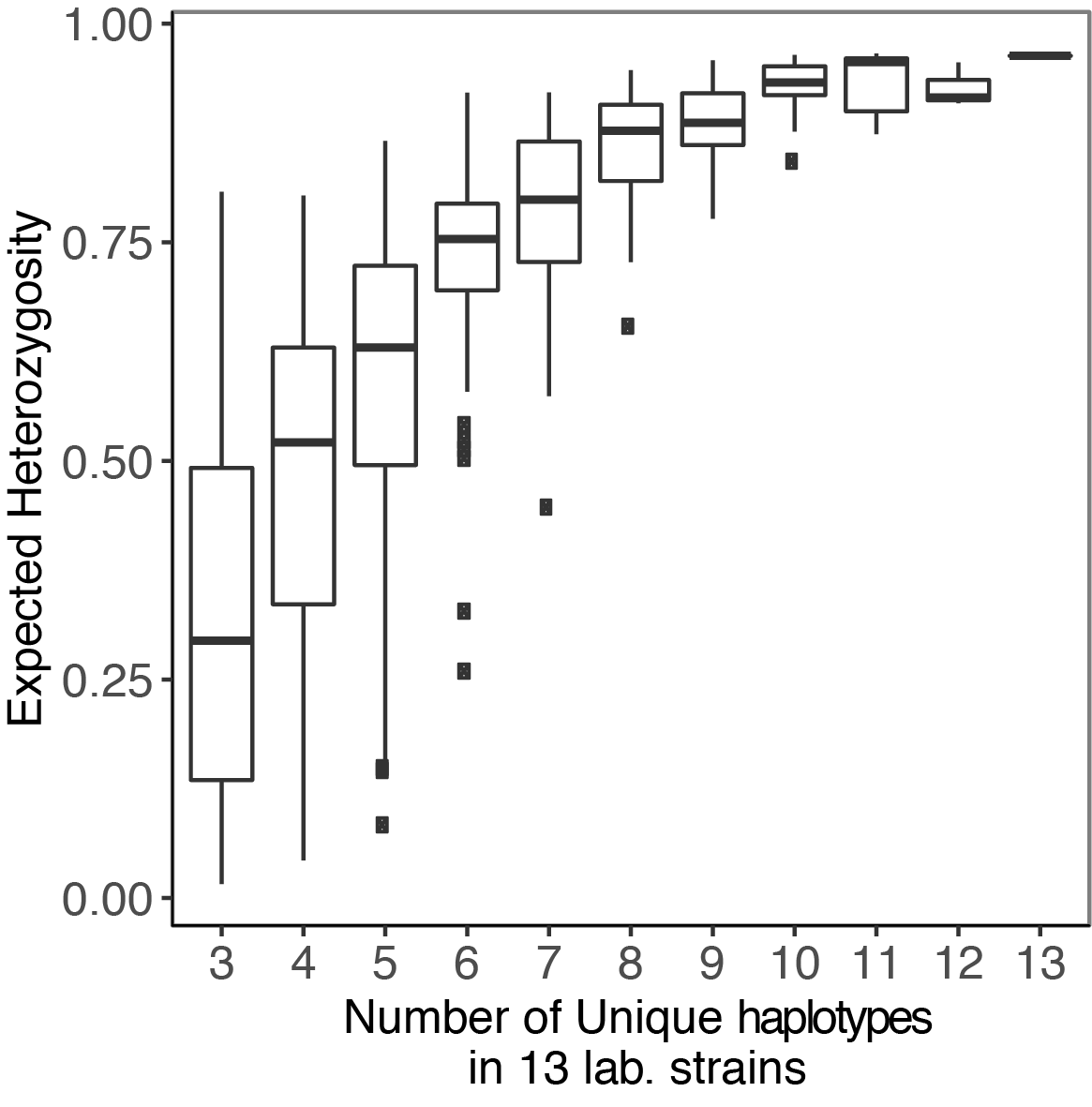
**

**Supplementary Figure 7. 13 Genomes Unique Seq Count to Global He**

Relationship between the number of unique haplotypes in 13 reference genomes and the expected heterozygosity of microhaplotypes in global parasite populations.


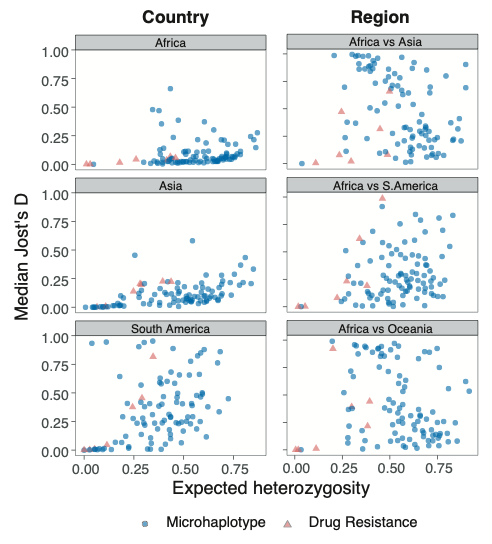


**Supplementary Figure 8 – Expected He to Jost’s D Across Regions**

Relationship between expected heterozygosity and genetic differentiation between countries within regions and between different regions.

**Supplementary Figure 9 – Mozambique Samples Panel Performance**

**A.** Boxplots summarize the coverage of microhaplotypes (>250 reads/target) and drug resistance targets by parasite density bins. **B.** Standard error in haplotype proportion between monoclonal and polyclonal replicates. The number of replicates per sample and the complexity of infections are shown. **C.** Distribution of multiplicity of infection in field samples from Mozambique.
