# Supplementary material for: Sensitive, Highly Multiplexed Sequencing of Microhaplotypes From the Plasmodium falciparum Heterozygome
Source: J Infect Dis. 2020 Aug 25;225(7):1227–37. doi: 10.1093/infdis/jiaa527 (PMC8974853; doi:10.1093/infdis/jiaa527)
Supplement: jiaa527_suppl_Supplementary_Methods [file jiaa527_suppl_supplementary_methods.docx]

**Supplementary Methods**

1. **Primer pool**

Primers were designed for 100 selected genomic regions of the microhaplotypes and drug resistance markers using the CleanPlex^®^ algorithm and synthesized by Paragon Genomics Inc, USA (Table A). A list of targets and primers is included in Supplementary Table 3.

**Table A. Description of primers**

| panel | Organism | Number of pools | Primer conc. | Number of targets | Average length | Library length |
| --- | --- | --- | --- | --- | --- | --- |
| PGD268 | Pf | 1 | 5X | 100 | 228bp | 300-400bp |

1. **Primary multiplexed PCR amplification**

Amplification of the 200-plex oligo pool was performed with some modifications of the CleanPlex® protocol (Paragon Genomics Inc, USA). Two sets of PCR conditions were optimized for low (i.e. ≤ 100 parasites per µL blood, condition “C1”) and high (i.e. > 100 parasites per µL blood, condition “C5”) parasite density dried blood spot samples. A two-step primary multiplex PCR was carried out in a 10 µL reaction containing 6 µL genomic DNA (Table B and C). Primary PCR product was diluted with 10 μl of 1X TE buffer and SPRI (Solid Phase Reversible Immobilization) bead purified using a sample:bead volume ratio of 1:1.3 and resuspended in 10μL 1X TE buffer after the final wash. Following bead purification, 10 μL of CleanPlex^®^ digest master mix was added and incubated for 10 min at 37°C and followed by SPRI bead purification using a sample:bead volume ratio of 1:1.3 and resuspended in 10μL (Table D).

**Table B. Master mix composition for the two PCR conditions**

| **Master mix for the multiple PCR** | | | | |
| --- | --- | --- | --- | --- |
| **C1 - condition** | |  | **C5 - condition** | |
| **Reagents*** | **Volume** |  | **Reagents*** | **Volume** |
| 5X mPCR Mix | 2μl |  | 5X mPCR Mix | 2μl |
| 5X mPCR Primer | 2μl |  | 5X mPCR Primer (1in 4 diluted) | 2μl |
| DNA Sample** | 6μl |  | DNA Sample** | 6μl |
| total | 10μl |  | total | 10μl |

*All reagents were supplied by Paragon Genomics Inc, CA, USA

**DNA was extracted from dried blood spot samples using Tween-Chelex extraction protocol.

**Table C. Cycling conditions**

| **Thermal Cycling Conditions** | | | | | | | | | |
| --- | --- | --- | --- | --- | --- | --- | --- | --- | --- |
|  | **C1 - condition** | | | |  | **C5 - condition** | | | |
| Step | Temp | Time | Ramping | Cycles |  | Temp | Time | Ramping | Cycles |
| Initial Denaturation | 95°C | 10 min | - | 1 |  | 95°C | 10 min | - | 1 |
| Denaturation | 98°C | 15 sec | 3°C/s | 10 cycles |  | 98°C | 15 sec | 3°C/s | 30 cycles |
| Annealing/  Extension | 60°C | 5 min | 2°C/s |  |  | 60°C | 5 min | 2°C/s |  |
| Hold | 10°C | ∞ |  |  |  | 10 °C | ∞ |  |  |

**Table D. Digestion reaction master mix**

| **Reagents*** | **Volume** |
| --- | --- |
| Nuclease-Free Water | 6μl |
| CP Reagent Buffer | 2μl |
| CP Digestion Reagent | 2μl |
| total | 10μl |

*All reagents were supplied by Paragon Genomics Inc, CA, USA

1. **Indexing PCR and pooling**

A secondary indexing PCR was performed in a 40 µL reaction containing 10 µL of bead purified digested product, 18μL of nuclease-free water, 8μL of 5X secondary PCR master mix, and 5 µL of 10 µM TruSeq i5/i7 barcode primers. PCR was carried out using the following cycling conditions: 10 min at 95°C, 13 cycles for high density samples (or 15 cycles for low density samples) of 15 sec at 98°C and 75 sec at 60°C. Samples were SPRI bead purified and quantified by capillary electrophoresis. All samples were proportionally pooled based on the estimated concentration of the capillary electrophoresis. The final library was bead purified, assessed for quality on the Bioanalzyer (Agilent technologies, Santa Clara, CA) and sequenced with 150bp paired end clusters on the Illumina NextSeq 550 instrument (Illumina, San Diego, CA, USA).

1. **Cost of Paragon multiplex PCR Protocol**

The total cost of multiplex PCR Protocol offered by [Paragon Genomics](https://www.paragongenomics.com/) including cost of primer design and cost of all necessary reagents to carry out protocol for 192 samples for 100 primer targets was $6,816.31 ($35.50 per sample cost).

1. **Cost of Amplicon vs WGS**

The whole genome sequence data (WGS) in this manuscript was generated by selective whole genome amplification (SWGA) off of dried blood spots. The rough cost estimate per sample for SWGA excluding sequencing is $32.00. Both the amplicon data and the WGS data was sequenced using an Illumina NextSeq machine using 150 paired-end using a high flow cell which generates approximately 400 million read pairs for a list cost of $2,780.00. For the amplicon data in order to generate 10,000 reads per target for 100 targets would require 1 million read pairs per sample while it would require 7.5 million read pairs for WGS data to achieve approximately 100x coverage across the *Plasmodium falciparum* genome. Therefore, the estimated cost per sample, including estimated sequencing costs, for the amplicon data would be $42.25 and for WGS would be $84.13.

1. **Targeted amplicon analysis**

The targeted amplicon data were analyzed using SeekDeep (v.2.6.6) [(Hathaway et al. 2018)](https://paperpile.com/c/aK34dE/zq1X). Briefly, Illumina pair-end sample files were demultiplexed on target primers and paired end reads stitched into a single read while filtering for per base quality and expected target lengths to create a FASTQ file for each target-sample pair. The SeekDeep qluster step was used to create haplotypes per sample per target by comparing sequences and collapsing on low per base quality errors and low frequency error to help eliminate sequencing and low-level PCR error. Each haplotype was given a within sample within target frequency based on the number of reads clustered together and haplotypes marked as possibly chimeric if there were two possible parent sequences at a frequency of at least twice as much as the possible chimeric haplotype. SeekDeep processClusters step then handled the final processing of the haplotypes by removing the marked possibly chimeric haplotypes. The final haplotypes for each target were then compared across samples to get population frequency calculations and to generate final population haplotypes. To help further remove possible artifact that was either introduced by PCR or by cross contamination between samples in either library prep or within the Illumina machine [(Costello et al. 2018)](https://paperpile.com/c/aK34dE/FFGw) several additional post processing filtering steps were performed during the SeekDeep processClusters step. Haplotypes that were either one SNP or one indel different from a within sample haplotype that was found with at least 10 times greater frequency were removed unless that haplotype was seen in another sample as a major haplotype. Haplotypes that only appeared in one sample and that were one difference off another haplotype within sample were also removed to reduce the detection of PCR jackpot events that happen early in PCR. Haplotypes were also removed if below a 2% frequency.
